# Supplementary material for: Paracrine Responses of Cardiosphere-Derived Cells to Cytokines and TLR Ligands: A Comparative Analysis
Source: Int J Mol Sci. 2023 Dec 8;24(24):17278. doi: 10.3390/ijms242417278 (PMC10743612; doi:10.3390/ijms242417278)
Supplement: Supplementary file 1 [file ijms-24-17278-s001.zip › Suppl.pdf]

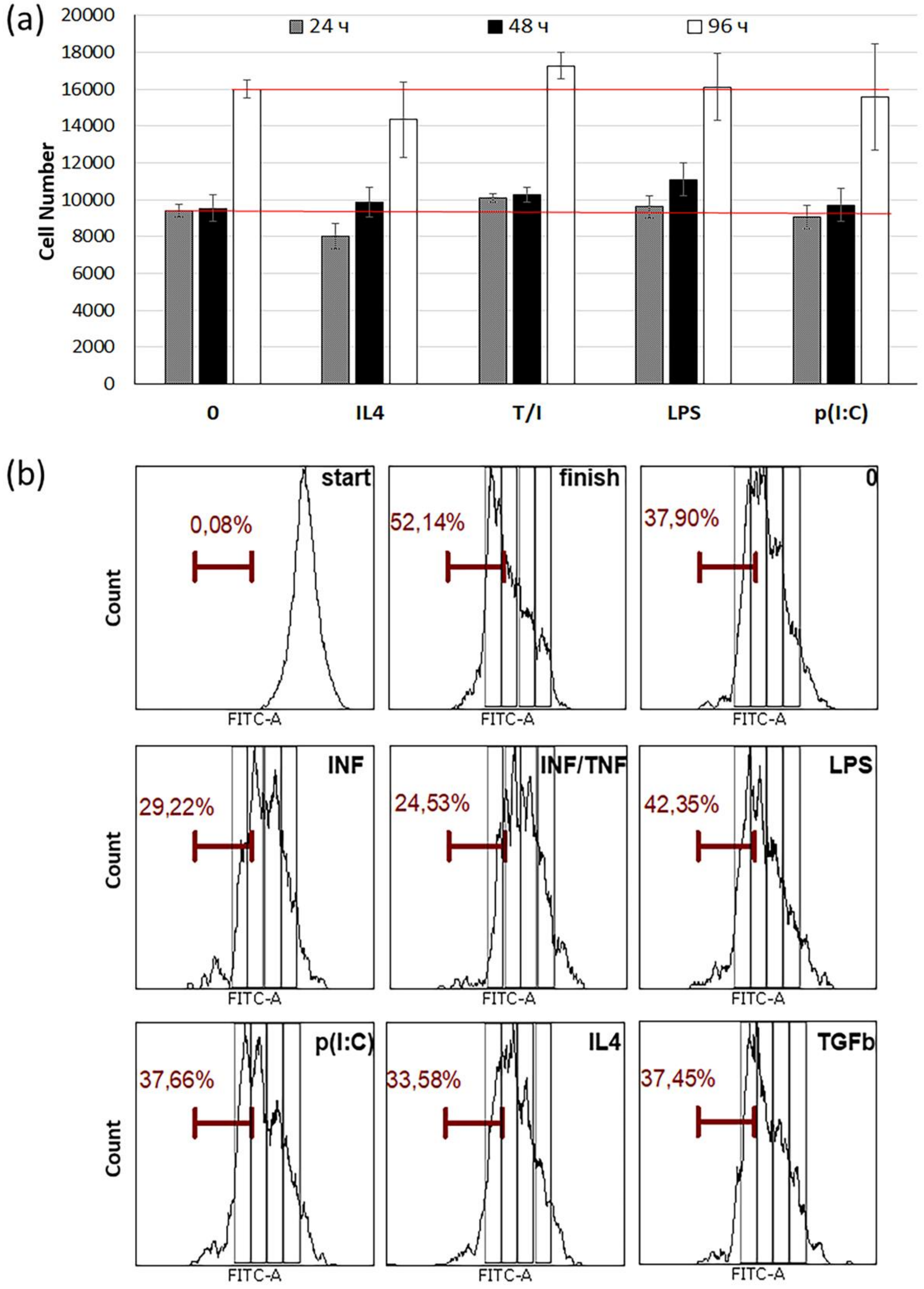

Figure S1. Effect of pre-incubation with cytokines and TLR-ligands on proliferation of CDC and PHA-activated lymphocytes. **(a)** Effect of pre-incubation for 48h with cytokines and TLR-ligands on CDC proliferation. Concentrations used for preliminary incubation: pIC: 1 µg/ml; IL4: 25 ng/ml; LPS: 10 ng/ml (LPS); TNF/INF: 20 ng/ml each of TNFa and INFg. "0"- medium – DMEM-F12/ITS/ 0,5% FBS. After pre-incubation for 48h with cytokines cells

were passaged and allowed to proliferate for additional 72h in assay media DMEM-F12/ITS/ 0,5% FBS. Data presented as mean ± standard deviation. Cell viability assessed via PrestoBlue$_{TM}$ staining.
(b) Flow cytometry analysis of CFSE-labeled lymphocyte proliferation. Representative flow cytometry histograms depicting the dilution of CMFDA fluorescence intensity in lymphocytes following stimulation with phytohemagglutinin-L for 3 days in the presence of CDCs treated with different factors. Start- labeled-lymphocyte before co-culture with CDCs. Finish - labeled-lymphocyte after 3 days without co-culture with CDCs. Bars indicate the assumed number of cells divisions. Markers indicate the percentage of proliferating CFSE-labeled lymphocytes at the last stage of division.

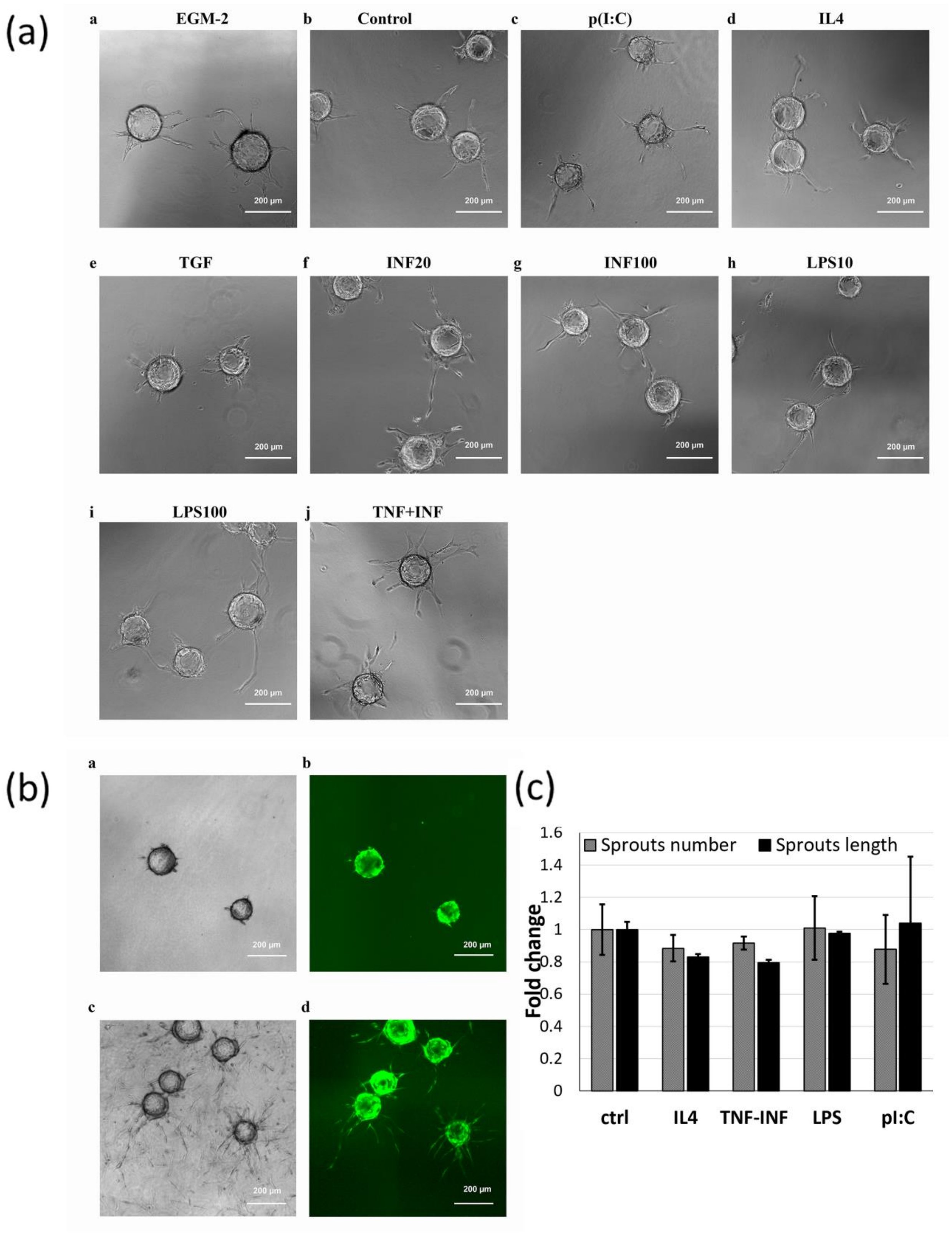

Figure S2. Representative images of fibrin gel bead assay. **(a)** HUVEC-coated bead were grown in the presence of CDCs conditioned media mixed with endothelial growth media EGM2 (1: 5) for 5 days. Images of gels were acquired in transmitted light channel with High-contents Imaging System Image ExFluorer (LCI, Korea). Scale bar represents 200 µm. **(b)** EC-coated microbeads were embedded in fibrin gel alone (a,b) or together with CDCs (c,d). Endothelial cells preliminarily were labeled with CellTracker Green CMFDA dye (Thermo Fisher). Photomicrographs were acquired at day 3 in transmitted light and green fluorescent channel; **(c)** Results from the fibrin gel angiogenesis bead assay. Cardiosphere-derived cells in fibrinogen solution were added to HUVEC-coated Cytodex beads and gel was allowed to polymerize. Endothelial sprout lengths were quantified after 5 days of co-culturing. Graphs represent the fold changes relative to untreated CDC ("CTRL").

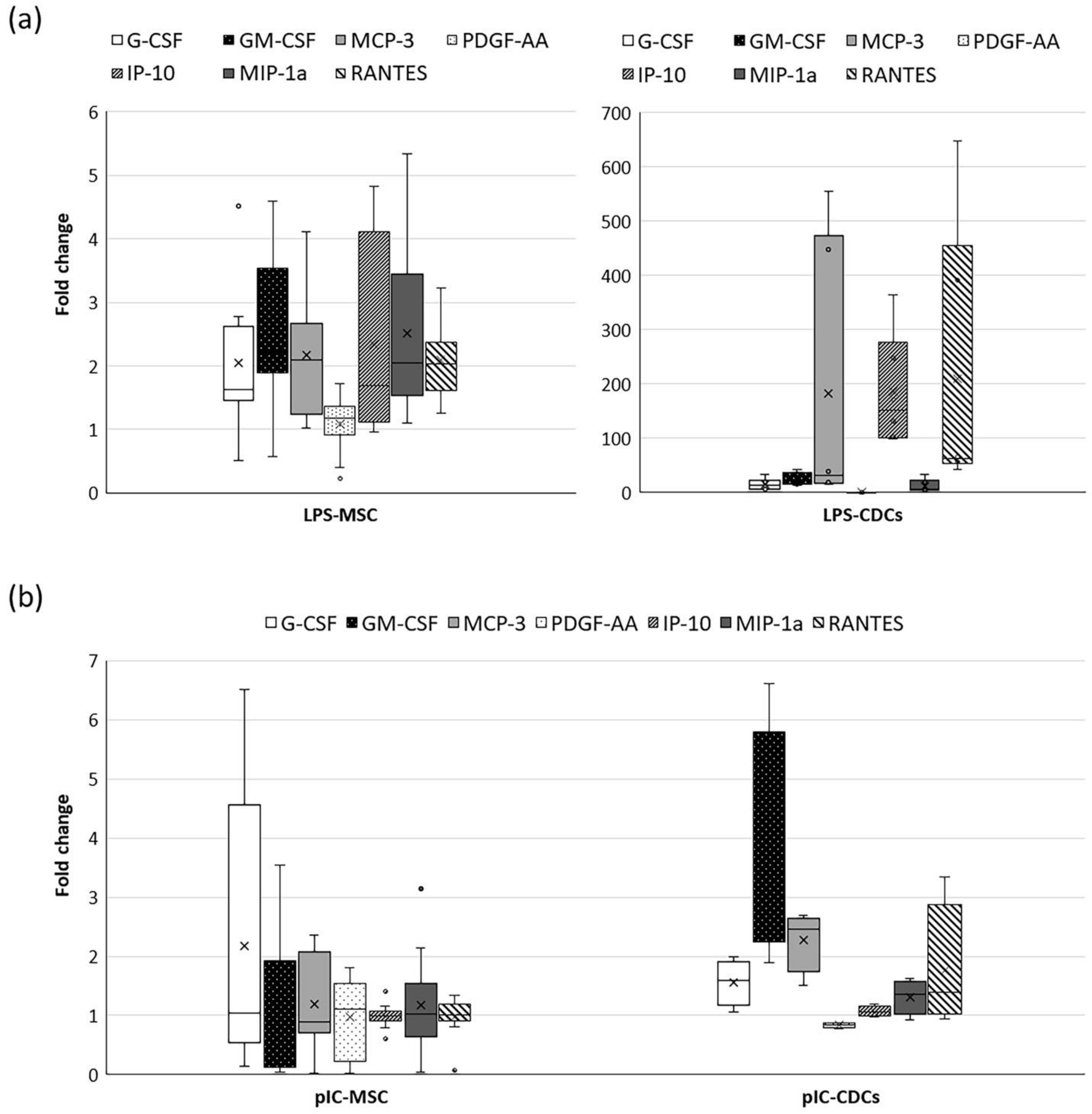

Figure S3. Comparative analysis of LPS and poly(I:C) treatment on cytokine secretion by MSCs and CDCs. Results of multiplex immunoassay (Magpix, HCYTMAG-60K-PX41) of the quantity of growth factors and cytokines in CDCs supernatants collected 24h after cell incubation with TLR-ligands for 1h. Expression changes are normalized to secretion levels of intact cell (without any stimulation). Box edges mark the 25th and 75th percentiles. Whiskers extend to 1.5 times the interquartile range from the 1st to the 3rd quartile; n=4.
