# Supplementary figures and images for: Paracrine Responses of Cardiosphere-Derived Cells to Cytokines and TLR Ligands: A Comparative Analysis
Source: Int J Mol Sci. 2023 Dec 8;24(24):17278. doi: 10.3390/ijms242417278 (PMC10743612; doi:10.3390/ijms242417278)

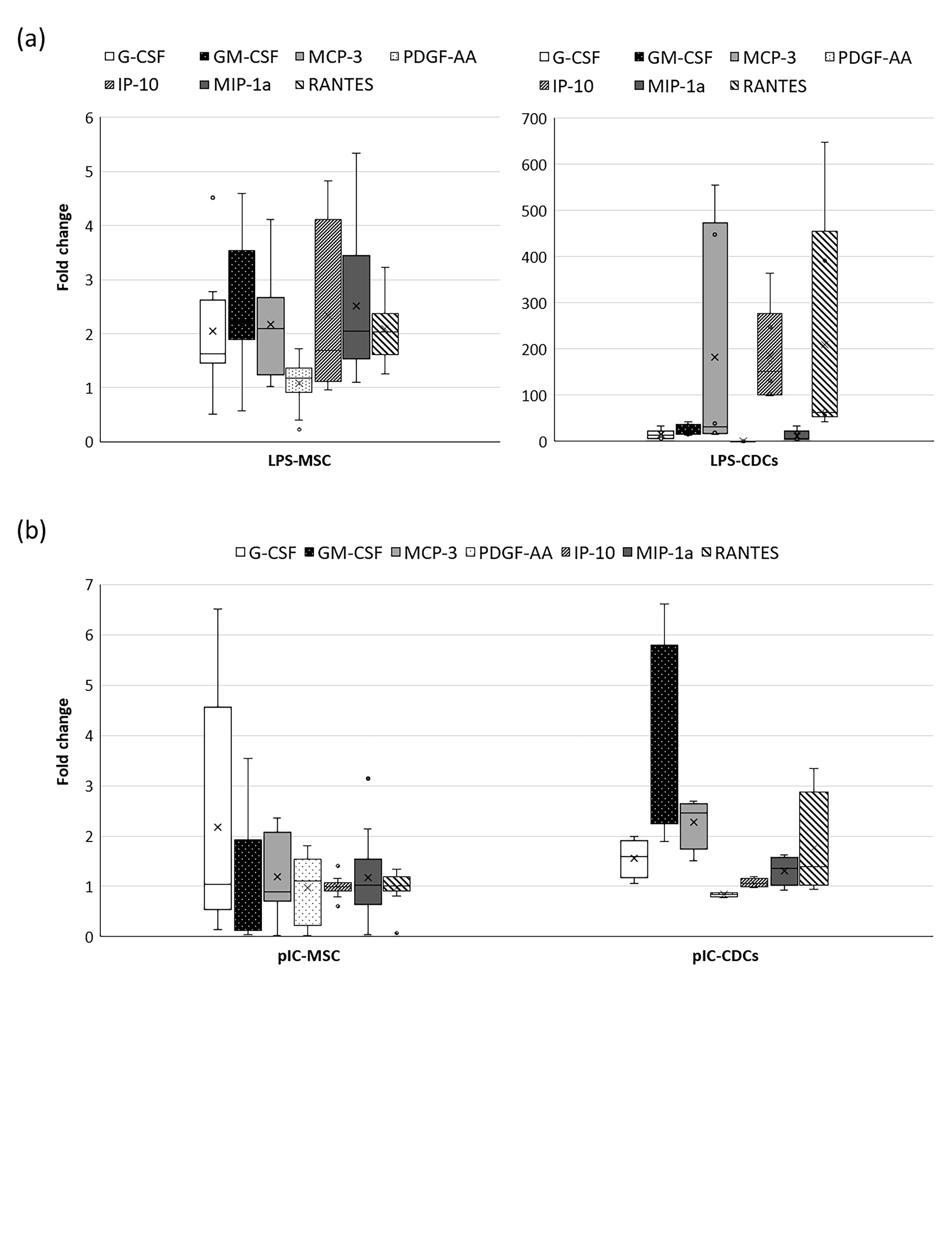

Supplement: Supplementary file 1 [file ijms-24-17278-s001.zip › sup3.tif]

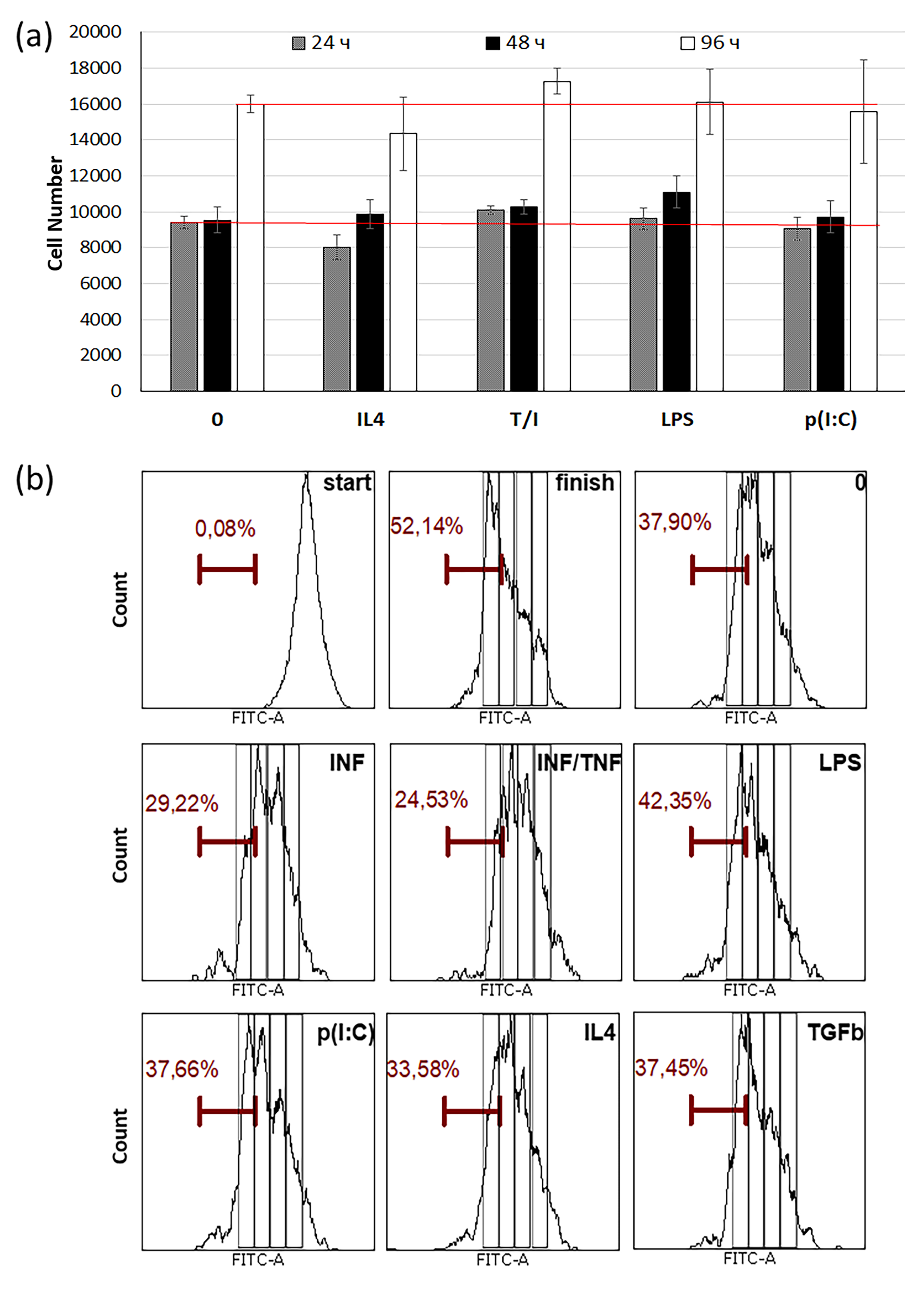

Supplement: Supplementary file 1 [file ijms-24-17278-s001.zip › supp1.tif]

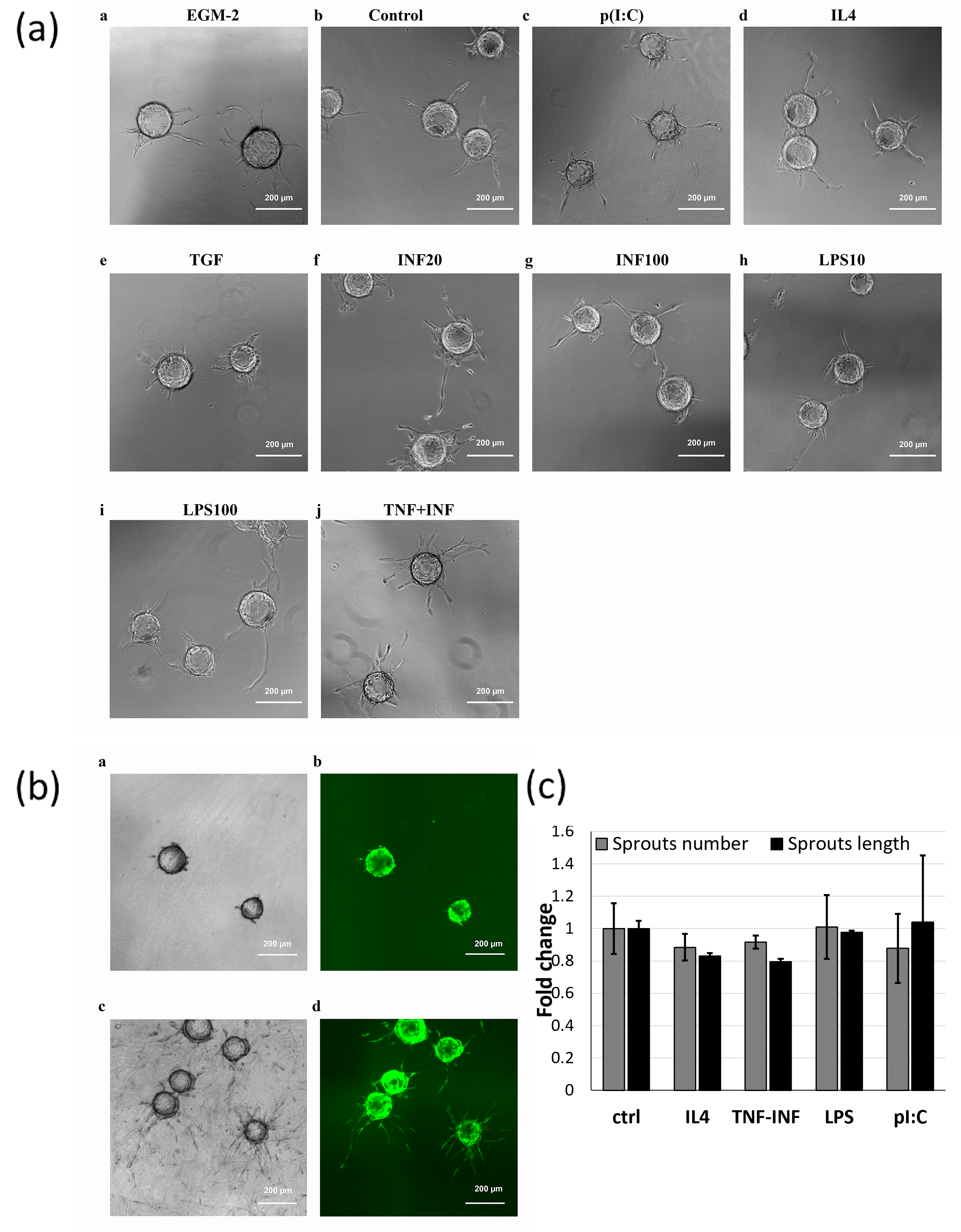

Supplement: Supplementary file 1 [file ijms-24-17278-s001.zip › supp2.tif]
